# A229 THE ASSOCIATION OF MENTAL HEALTH DISORDERS WITH FATIGUE AND PHYSICAL ACTIVITY IN PERSONS WITH IBD

**DOI:** 10.1093/jcag/gwae059.229

**Published:** 2025-02-10

**Authors:** C L Dolovich, S Chochinov, G Ly, A Oketola, S Narvey, S Larance, M Raman, S Webber, C N Bernstein

**Affiliations:** Internal Medicine, University of Manitoba Max Rady College of Medicine, Winnipeg, MB, Canada; University of Manitoba Max Rady College of Medicine, Winnipeg, MB, Canada; University of Manitoba, Winnipeg, MB, Canada; University of Manitoba, Winnipeg, MB, Canada; Internal Medicine, University of Manitoba Max Rady College of Medicine, Winnipeg, MB, Canada; University of Manitoba, Winnipeg, MB, Canada; University of Calgary, Calgary, AB, Canada; University of Manitoba College of Rehabilitation Sciences, Winnipeg, MB, Canada; University of Manitoba Max Rady College of Medicine, Winnipeg, MB, Canada

## Abstract

**Background:**

Fatigue is one of the most common symptoms among persons with inflammatory bowel disease (IBD) that impairs their quality of life. Fatigue acts as one of the greatest barriers of physical activity (PA) participation in adults with chronic disease, while PA is a strong contributor to improving fatigue symptoms.

**Aims:**

The aim of this study was to assess the relationship between fatigue and PA in patients with IBD. A secondary aim was to assess the impact of mental health and disease activity on both PA and fatigue outcomes.

**Methods:**

Participants from the University of Manitoba IBD Research Registry (n=2740) were invited to complete a cross-sectional survey to understand how persons with IBD experience PA and exercise. The international Physical Activity questionnaire (IPAQ) was used to define low vs. moderate/high levels of PA. The Modified Fatigue Impact Scale (MFIS) was used to define participants as Fatigued (MFIS ≥38) vs. non-fatigued (MFIS<38). Mental health and disease activity were defined based on Generalized Anxiety Disorders-7 (GAD), Patient Health Questionnaire (PHQ-9) and Inflammatory Bowel Disease Symptom Inventory (IBDSI). Descriptive statistics, bivariate and logistic regression analysis were used to assess the relationships defined in the aims.

**Results:**

Among 1257 (46%) who completed the survey, there was 1075 (86%) and 1119 (89%) completed data for fatigue and PA, respectively. 93/706 (13%) of persons participated in low PA and were fatigued. Older patients were both less fatigued & less active than younger patients (median IQR: 62 (53-70) vs 60 (50-68), p=0.045). Nonactive to low physically active and high fatigued patients were more likely to have an education of high school degree or less (39% vs 25%, p<0.01), an income less than $50,000 (32% vs 14%, p<0.0001), not in a relationship (27% vs 17%, p<0.01), have an ostomy bag (14% vs 7%, p=0.02), and ever smoked (19% vs 6%, p<0.0001) compared to moderate/high physically active non-fatigued patients. The odds of low PA and high fatigue were greater for persons with anxiety [aOR 95%CI; 3.0 (1.2-7.2)], depression [23.5 (10.8-53.6)], and active disease [3.97 (1.9-8.3)].

**Conclusions:**

Patients with IBD are more likely to be participating in low levels of PA & be fatigued when they have a lower education & income, not in a relationship, have an ostomy bag and are a current or previous smoker. The odds of low PA and fatigue are up to 23 times greater for persons with a mental health disorder. New insights on PA and fatigue provide an impetus for PA intervention as it may help alleviate fatigue symptoms and enhance quality of life among persons with IBD.

**Funding Agencies:**

CAGn/a

